# Identification and Differential Expression of MicroRNAs during Metamorphosis of the Japanese Flounder (*Paralichthys olivaceus*)

**DOI:** 10.1371/journal.pone.0022957

**Published:** 2011-07-27

**Authors:** Yuanshuai Fu, Zhiyi Shi, Minglin Wu, Junling Zhang, Liang Jia, Xiaowu Chen

**Affiliations:** Key Laboratory of Exploration and Utilization of Aquatic Genetic Resources, Shanghai Ocean University, Ministry of Education, Shanghai, People's Republic of China; Beckman Research Institute of the City of Hope, United States of America

## Abstract

**Background:**

MicroRNAs (miRNAs) are a class of endogenous small non-coding RNAs of 20–25 nucleotides that play a key role in diverse biological processes. Japanese flounder undergo dramatic metamorphosis in their early development. The metamorphosis is characterized by morphological transformation from a bilaterally symmetrical to an asymmetrical body shape concomitant with extensive morphological and physiological remodeling of organs. So far, only a few miRNAs have been identified in fish and there are very few reports about the Japanese flounder miRNA.

**Methodology/Principal Findings:**

Solexa sequencing technology was used to perform high throughput sequencing of the small RNA library from the metamorphic period of Japanese flounder. Subsequently, aligning these sequencing data with metazoan known miRNAs, we characterized 140 conserved miRNAs and 57 miRNA: miRNA* pairs from the small RNA library. Among these 57 miRNA: miRNA* pairs, twenty flounder miRNA precursors were amplified from genomic DNA. We also demonstrated evolutionary conservation of Japanese flounder miRNAs and miRNA* in the animal evolution process. Using miRNA microarrays, we identified 66 differentially expressed miRNAs at two metamorphic stages (17 and 29 days post hatching) of Japanese flounder. The results show that miRNAs might play a key role in regulating gene expression during Japanese flounder metamorphosis.

**Conclusions/Significance:**

We identified a large number of miRNAs during flounder metamorphosis, some of which are differentially expressed at two different metamorphic stages. The study provides an opportunity for further understanding of miRNA function in the regulation of flounder metamorphosis and gives us clues for further studies of the mechanisms of metamorphosis in Japanese flounder.

## Introduction

MicroRNAs (miRNAs) are endogenous, ∼22 nucleotides (nt), small non-coding RNA molecules that regulate gene expression by complementary binding to the 3′ untranslated region (UTR) of target messenger RNAs (mRNAs) and causing mRNA cleavage or translation blockage [1]. MiRNAs play a key role in diverse biological processes such as organ development, cell proliferation, tumorigenesis, fat metabolism, behavior and embryogenesis [1]–[6]. Furthermore, abnormal expression of miRNA may result in disease, dramatic phenotype variation, or death [7]. Recent studies have suggested that miRNA may be an inducible factor to increase organismal complexity through their function in regulating gene expression [8]–[11]. The first characterized endogenous miRNAs were lin-4 and let-7, both of which were found to act in the pathway controlling the timing of larval development in *C. elegans* [12]–[13]. Since then, a number of miRNAs have been discovered in different animal species. MiRNAs are currently estimated to comprise 1% to 5% of animal genes and collectively regulate up to 30% of genes, making them key regulators [14].

Most of conserved miRNAs have been discovered by traditional identification method. However, species/family non-conserved miRNAs are seldom identified by the traditional method, because they are often expressed at a lower level than conserved miRNAs [15]. With widespread application of high-throughput sequencing technologies, both conserved and non-conserved miRNAs have been identified from diverse organism such as amphioxus (*Branchiostoma lanceolatum*), locust (*Locusta migratoria*), silkworm (*Bombyx mori*), red alga (*Porphyra yezoensis*) and barrel medic (*Medicago truncatula*) [15]–[19].

Japanese flounder (*Paralichthys olivaceus*) is one of the most important marine economic fish. It undergoes metamorphosis by changing its body from larval to juvenile forms. The metamorphosis is prominently characterized by morphological transformation from a bilaterally symmetrical to an asymmetrical body shape and accompanied by extensive morphological and physiological remodeling of tissues/organs [20]–[21]. These structural and functional changes are linked to transcriptional or post transcriptional regulation of gene expression. Previous studies showed that some miRNAs identified in metamorphic species play an important role in regulating the biological process of metamorphosis [17]–[18], [22]–[24]. Given that miRNAs play key roles in regulating gene expression during animal development, studies of miRNAs during Japanese flounder metamorphosis are peculiarly useful. Fish represent approximately half of all vertebrate species, and previous researches have indicated that many fish miRNAs are identified from rainbow trout (*Oncorhynchus mykiss*), zebrafish (*Danio rerio*), fugu (*Takifugu rubripes*), green pufferfish (*Tetraodon nigroviridis*) and medakafish (*Oryzias latipes*), whose genome sequence data are available [25]–[28]. Though we are currently short of substantial genome sequence data of Japanese flounder, it is feasible to identify miRNAs by means of alignment with miRbase 13.0 and expressed sequence tags (ESTs).

Previously, Xie *et al.* identified 23 conserved miRNAs at the metamorphic stage of Japanese flounder by traditional cloning method, indicating that miRNAs play an important role in larva-to-juvenile metamorphosis [29]. In our study, we used a high-throughput Solexa sequencing method (Illumina Genome Analyzer) to further identify Japanese flounder miRNAs. We obtained 140 conserved miRNAs and 57 miRNA*s during Japanese flounder metamorphosis. Subsequently, we examined expression patterns of these miRNAs at two metamorphic stages by miRNA microarray and identified 66 differentially expressed miRNAs between two metamorphic stages. These findings are conducive to better understanding of miRNA roles in regulating diverse biological processes during metamorphosis.

## Results

### Solexa sequencing of small RNAs

**Figure 1:**
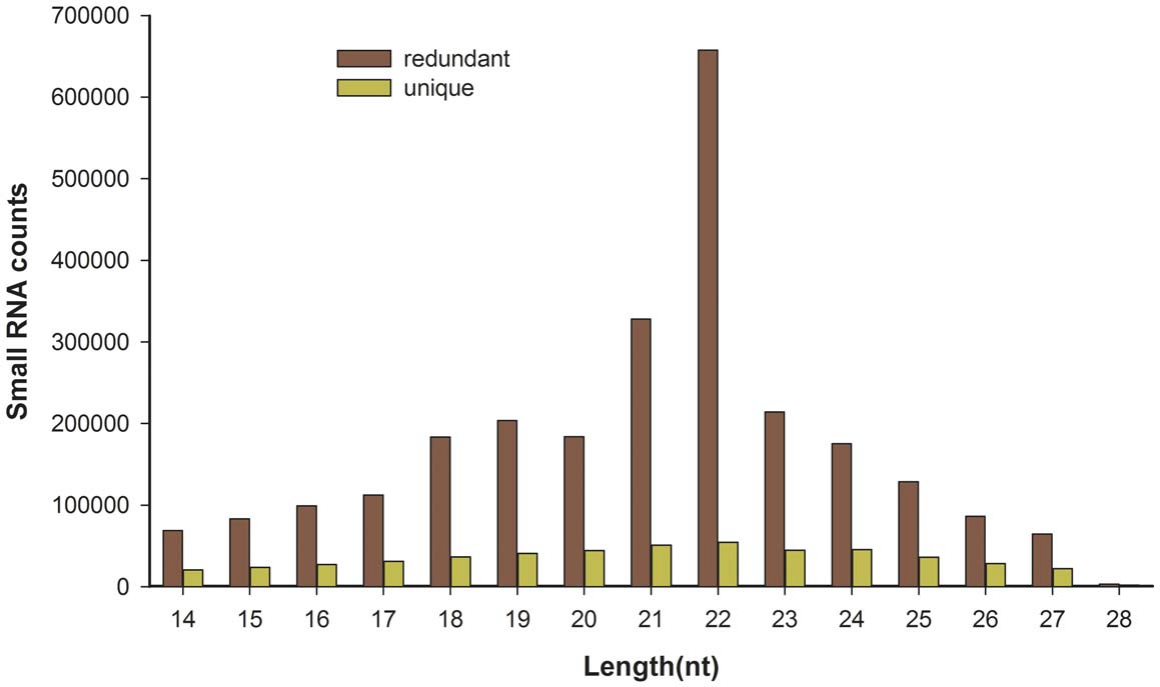
Size distribution of sequenced small RNAs. The redundant represents the number of all sequences, and the unique represents the number of unique sequences. nt, nucleotides.

To identify flounder miRNAs during metamorphosis, a small cDNA library that was generated from a mixture of total RNAs from nine metamorphic stages was subjected to high-throughput sequencing by the Illumina platform. After the raw sequences were subjected to Illumina Pipeline filter provided by vendor (Solexa 0.3), a total of 3108333 reads, representing 931491 unique sequences, were obtained from the small cDNA library and uploaded to Gene Expression Omnibus [GEO:GSE25995]. We analyzed the length distribution of small RNAs (14–28 nt long) sequenced by Solexa sequencing (Figure 1). After discarding 1567405 reads of sample sequences, junk sequences, simple sequences, sequences longer than 26 nt and shorter than 15 nt and sequences with fewer than 3 copies, 1540928 reads remained for analysis. After comparing the small RNA sequences with Japanese flounder mRNAs, RFam and Repbase, 431953 reads from mRNA, rRNA, tRNA and snoRNA were removed, as well as repeat sequences. The remaining 1108975 reads were retained for miRNA analysis.

### Conserved miRNAs and miRNA*s

To identify the known miRNAs in Japanese flounder, we compared our dataset to known metazoan miRNAs (miRNA precursors and mature miRNAs) in miRBase 13.0 (http://www.mirbase.org/) by miRAlign. MiRAlign is a computational method that is capable of detecting new miRNAs based on both sequences and structure alignment [30]. Among the screened 1108975 reads, 588793 reads that represent 2859 unique sequences were mapped to known metazoan miRNAs in miRBase 13.0. Allowing one mismatch between sequences, we then identified 140 conserved miRNAs from 394842 reads, 133 belonging to 84 families and 7 not belonging to any family (Table S2). These miRNAs share the same ‘seed’ regions (the two to eight bases of the 5′ region important for target recognition) [31] in Japanese flounder as other metazoan. Furthermore, from the 2859 unique sequences, we obtained 57 duplex-like miRNA: miRNA* pairs in which the mature miRNAs and miRNA*s respectively aligned to the 5′ end or 3′ end region, of known miRNA precursors in the library. In other words, 57 miRNA* candidates were found from 9734 reads during flounder metamorphosis. We compared the 57 miRNA* candidates with conserved mature miRNA*s in miRBase and identified 47 miRNA* allowing three mismatches. Another 10 miRNA*s that have much more mismatches between sequences need to be further validated (Table S3). Such results show that miRNA*s are less stable than miRNAs during species evolution [32]. Among the 140 conserved miRNAs and 57 miRNA*s, nine miRNAs (mir-1, -206a, -192, -203, -22a, -122, -92a, let-7a, and let-7d) had more than ten thousand reads, indicating they are highly expressed during flounder metamorphosis (Figure S1 and Figure S2). We also found that 10 members of the let-7 family (including let-7a, -7b, -7c, -7d, -7e, -7f, -7g, -7h, -7i and -7j) were expressed, implying that the let-7 family might play an important role in regulating Japanese flounder metamorphosis.

**Figure 2:**
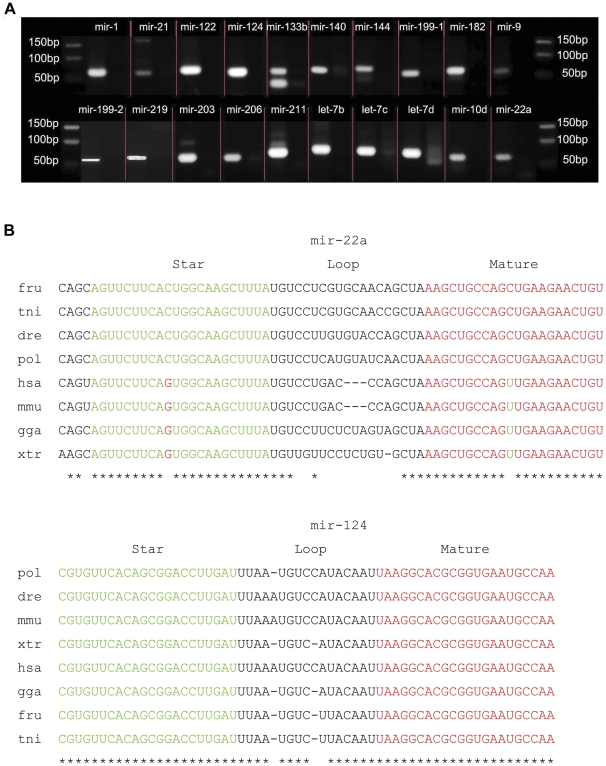
Conservation of miRNAs in the Japanese flounder. (A) Electrophoretic analysis of PCR products amplified from flounder genomic DNA by the primer pairs designed on the basis of miRNA-5p and miRNA-3p based on similarity to miRNA stem-loop sequences in metazoon. For each miRNA, the left lane is the test sample and the right lane is the negative control. (B) Sequence alignment analysis of two miRNA precursors that have both conserved star and mature sequences. The alignment of mir-22a and mir-124 in different species shows high conservation of their mature miRNA and miRNA*. The green nt represent miRNA* sequence and the red represent mature miRNA sequence. The asterisks indicate the conserved sites among these species. dre, *Danio rerio*; fru, *Takifugu rubripes*; gga, *Gallus gallus*; xtr, *Xenopus tropicalis*; has, *Homo sapiens*; mmu, *Mus musculus*; pol, *Paralichthys olivaceus*; tni, *Tetraodon nigroviridis*; bp, base pairs; nt, nucleotides.

We used a PCR-based method to verify the relationship between a miRNA and its miRNA*. If miRNA and its miRNA* came from the same precursors, a 55–70 nt should be amplified from the genomic DNA [17]. We amplified 55–70 nt products of twenty miRNAs that were at random selected from 57 miRNA: miRNA* species (Figure 2A). Although there are faint bands and multiple bands in some of the lanes, which are potentially nonspecific amplification products and primer dimer, we thought that these bands have little effect on the target fragment of 55–70 nt, so we purified and sequenced the 55–70 nt PCR fragment. These sequencing results confirmed the matches between mature miRNAs and miRNA*s (Table 1). Subsequently, we predicted the secondary structures of these amplified sequences by mfold [33], and found that all 20 sequences could be properly folded into the typical hairpin structure (Figure S3). The results indicated that these 20 miRNA: miRNA* pairs respectively came from the same precursor. The data also demonstrated that, in addition to conservation of mature miRNAs, some flounder miRNA*s may be highly conserved in different species (Figure 2B).

**Table 1:** Mature and precursor sequences of conserved miRNA in Japanese flounder.

miRNA name	Sequences of the precursors (5′-3′)
pol-mir-1	*ACAUACUUCUUUAUAUGCCCAUA*UGAACAAGAGCAACUA**UGGAAUGUAAAGAAGUAUGUAU**
pol-let-7b	**UGAGGUAGUAGGUUGUGUGGUU**UCAGGGUUGUGAUUUUACCCCAUCAGGAGCUAA*CUAUACAACCUACUGCCUUCC*
pol-let-7c	**UGAGGUAGUAGGUUGUAUGGUU**UGUGGGAUGGAGUAAAUCCUACUCAGGGGAUAA*CUAUACAACCUACUGCCUUCC*
pol-let-7d	**UGAGGUAGUUGGUUGUAUGGUU**UCGCAUAAUAAACAGCACGGAGAUAA*CUGUACAACCUUCUAGCUUUCC*
pol-mir-9	**UCUUUGGUUAUCUAGCUGUAUGA**GUUUUAAUUUCA*UAAAGCUAGAGAACCGAAAGUA*
pol-mir-10d	**UACCCUGUAGAACCGAAUGUGU**GUGAUGCAACCACAGUCAC*AGAUUCGAUUCUAGGGGAGUAU*
pol-mir-21	**UAGCUUAUCAGACUGGUGUUGG**CUGUUUAGAUUGCAAGG*CGACAACAGUCUGAAGGCUGUC*
pol-mir-22a	CAGC*AGUUCUUCACUGGCAAGCUUUA*UGUCCUCAUGUAUCAACUA**AAGCUGCCAGCUGAAGAACU**GU
pol-mir-122	CUG**UGGAGUGUGACAAUGGUGUUUG**UGUCCUGUCUAUCA*AACGCCAUUAUCACACUAUAUA*GC
pol-mir-124	*CGUGUUCACAGCGGACCUUGAU*UUAAUGUCCAUACAAU**UAAGGCACGCGGUGAAUGCCAA**
pol-mir-133b	*GCUGGUCAAACGGAACCAAGU*CAGGUGUUUCUGUGAGG**UUUGGUCCCCUUCAACCAGCU**A
pol-mir-140	**CAGUGGUUUUACCCUAUGGUAG**GUGACAUCAUGCUGUUCU*ACCACAGGGUAGAACCACGGAC*
pol-mir-144	GCGG*GGAUAUCAUCUUAUACUGUAAGU*UUAUUAUAGAGACAC**UACAGUAUAGAUGAUGUACUAU**CCCG
pol-mir-182	**UUUGGCAAUGGUAGAACUCACA**CUGGUGAGGUAGAUGGAUCCGG*UGGUUCUAGACUUGCCAACUA*
pol-mir-199-1	**CCCAGUGUUCAGACUACCUGUU**CAUUGUCAUACUGGUGU*ACAGUAGUCUGCACAUUGGUUA*
pol-mir-199-2	**CCCAGUGUUCAGACUACCUGUU**CAGGAAGUAGUGGUUGU*ACAGUAGUCUGCACAUUGGUUA*
pol-mir-203	*AGUGGUUCUCAACAGUUCAACAG*UUCUUAGAGAAAAUU**GUGAAAUGUUUAGGACCACUUG**
pol-mir-206a	*ACAUGCUUCCUUAUAUCCCCAU*AUUCAUACAGCACUUA**UGGAAUGUAAGGAAGUGUGUGG**
pol-mir-219	**UGAUUGUCCAAACGCAAUUCUU**GUAUCACUUGUCUGUAUCUA*GGAGUUGUGGAUGGACAUCACG*
pol-mir-221	UGA*ACCUGGCAUACAAUGUAGAUUU*CUGUGUGUCAGUCUAC**AGCUACAUUGUCUGCUGGGUUU**CA

The bold letters represent the mature miRNAs squences, the italic ones represent its miRNA* sequences.

To identify non-conserved miRNAs in the Japanese flounder, we compared the sequencing data with pleuronectoidei ESTs (see Materials and methods) and obtained three miRNA candidates mapped to pleuronectoidei ESTs. Subsequently, expression of the three candidates wasn't detected at the metamorphic stage of Japanese flounder by Northern hybridization and Quantitative real-time PCR.

### Validation of Japanese flounder miRNAs expression

**Figure 3:**
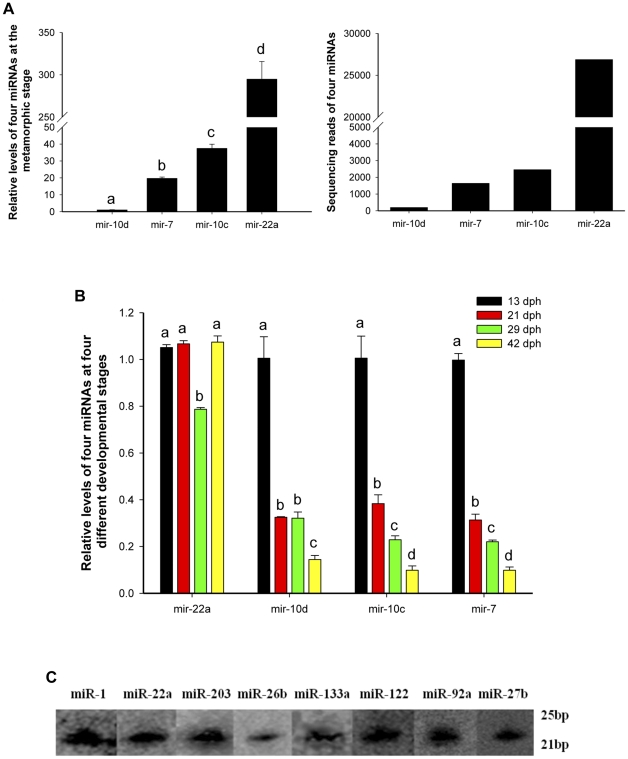
Validation of miRNAs by Quantitative real-time PCR and Northern hybridization. (A) Lift side: Expression levels of four miRNAs (mir-10d, -7, -10c, and -22a) at the flounder metamorphic stage. Different letters indicate significant differences between the corresponding metamorphic stages, *p*<0.05. Right side: Sequencing reads of four miRNAs by Solexa sequencing. (B) Relative expression levels of four miRNAs (mir-22a, -10d, -10c, and -7) at four different developmental stages (13, 21, 29, and 42 dph) of Japanese flounder, respectively. Different letters indicate significant differences between the corresponding metamorphic stages in four various miRNAs, *p*<0.05. (C) Expression analysis of eight miRNAs by Northern hybridization at the flounder metamorphic stage. dph, day post hatching; bp, base pairs.

To validate the newly identified Japanese flounder miRNAs, we conducted stem-loop Quantitative real-time PCR assays [34]–[35] from the same RNA preparation used for the Solexa sequencing. Among 140 conserved miRNAs, we randomly selected four miRNAs (mir-7, -10d, -10c and -22a, with 1635, 192, 2453 and 26871 reads, respectively) and examined their expression patterns by Quantitative real-time PCR in a total RNA pool from nine metamorphic stages of Japanese flounder. The results showed that the expression levels of these miRNAs were concordant with their relative reads of Solexa sequencing (Figure 3A). We also examined their expression at four different developmental stages (13, 21, 29, 42 dph) and found that they were differentially expressed (Figure 3B). In addition, Using Northern hybridization, we randomly examined expression levels of eight miRNAs (mir-1, -22a, -203, -26b, -133a, -122, -92a, and -27b) from the newly identified Japanese flounder miRNAs at the metamorphic stage (Figure 3C). These results showed that the above-mentioned miRNAs are authentic miRNAs and that some miRNAs are differentially expressed at different developmental stages of Japanese flounder.

Moreover, we detected the expression of the newly identified miRNAs in two metamorphic stages (17 and 29 dph) of Japanese flounder by miRNA microarray and have uploaded the raw data to Gene Expression Omnibus [GEO:GSE27986]. The results showed that 102 conserved miRNAs are identified by microarray (Figure S4). Nearly 61% of the miRNAs (86 out of 140) and 23% of the miRNA*s (16 out of 57) could be detected by microarray, and most undetected miRNAs and miRNA*s had low expression levels. The results further confirmed the authenticity of the miRNAs identified by Solexa sequencing in Japanese flounder.

### Differential expression of miRNAs in the two stages

**Figure 4:**
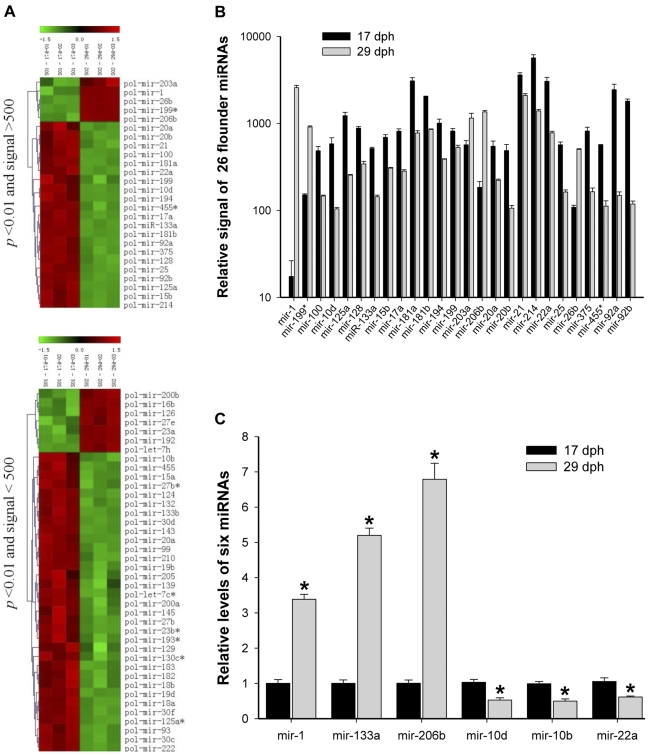
Differential expression levels of miRNAs in the 17 and 29 dph. (A) Differential expression of miRNAs at two metamorphic stages (17 and 29 dph) by hierarchical clustering. Upside: Differential expression of 26 miRNAs at the two stages (*p*<0.01 and signal >500). Downside: Differential expression of 40 miRNAs at the two stages (*p*<0.01 and signal <500). Red indicates that a gene is highly expressed at that stage, whereas green indicates the opposite. The absolute signal intensity ranges from −1.5 to +1.5, with corresponding color changes from blue to green, yellow and red. The signal of expression was detected by microarray with three probe repeats. (B) Relative detectable signals of 26 differentially expressed miRNAs (*p*<0.01 and signal >500) at two metamorphic stages (17 and 29 dph) by microarray. The signals are average (of three replicates) absolute intensity. (C) Relative expression levels of six miRNAs (mir-1, -133a, -206b, -10d, -10b and -22a) at two metamorphic stages (17 and 29 dph) by Quantitative real-time PCR. ^*^ *p*<0.05 means a statistically significant difference between this miRNA levels at 17 and 29 dph.

Clarification of the molecular mechanism of Japanese flounder metamorphosis is of great importance for the cultivation of Japanese flounder. Thus, it is interesting to uncover the differential expression profile of Japanese flounder miRNAs by miRNA microarrays. Among these 102 miRNAs identified by microarray, 66 flounder miRNAs were differentially expressed by comparing with miRNA expression patterns at two metamorphic stages (17 and 29 dph), as shown by microarray analysis (Table S4), including 26 conserved miRNAs (*p*<0.01 and Signal >500, Figure 4A and Figure 4B) and 40 conserved miRNAs (*p*<0.01 and Signal <500, Figure 4A), which suggested miRNAs may play an important role in regulating diverse biological processes during flounder metamorphosis. For example, expression levels of mir-1 and mir-206b at 17 dph were significantly lower than those at 29 dph, while expression levels of mir-10d and mir-22a at 17 dph were significantly higher than those at 29 dph. These results indicated that miRNAs might be involved in the regulation of Japanese flounder metamorphosis.

To confirm the microarray results, we randomly selected six miRNAs (mir-1, -133a, -206b, -10d, -10b and -22a) from 66 differentially expressed miRNAs and performed Quantitative real-time PCR analysis (Figure 4C). Five miRNAs showed similar expression patterns as those revealed by our microarray analysis. For unknown reasons, the expression levels of mir-133a were inconsistent with the microarray results.

### The phylogenetic evolution of miRNAs

**Figure 5:**
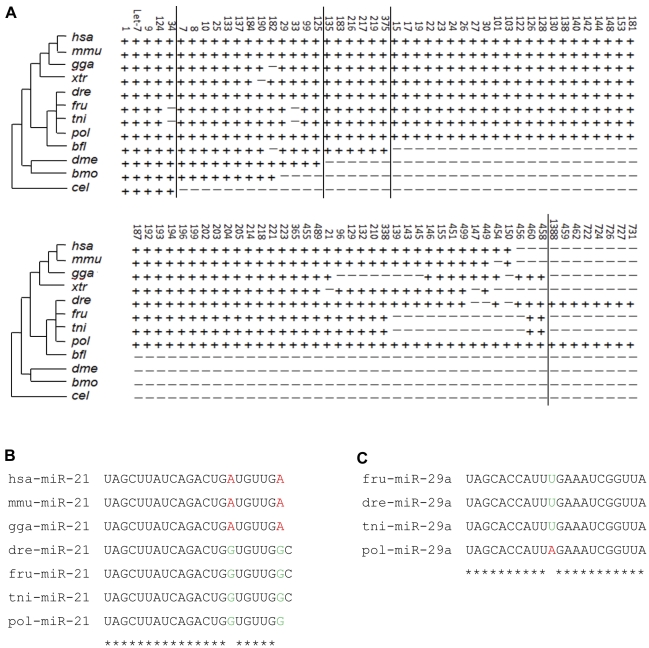
Phylogenetic evolution of Japanese flounder conserved miRNA families. (A) Phylogenetic distribution of Japanese flounder conserved miRNA families. A plus (+) symbol represents this miRNA family exists in the species named on the left, and a minus (−) symbol means it is absent in that species. (B) A clade-specific conserved miRNA based on sequence substitutions. The green nucleotides indicate the positions that are the same among the other vertebrates but different from fish, which are shown in red. (C) A flounder-specific conserved miRNA based on sequence substitution. The red nucleotide shows a Japanese flounder-specific position that is different from any other fish. Asterisks indicate the conserved sites among fish. bfl, *Branchiostoma floridae*; bmo, *Bombyx mori*; cel, *Caenorhabditis elegans*; dme, *Drosophila melanogaster*. has-miR-21, *Homo sapiens* microRNA 21; mmu-miR-21, *Mus musculus* microRNA 21; gga-miR-21, *Gallus gallus* microRNA 21; dre-miR-21, *Danio rerio* microRNA 21; fru-miR-21, *Takifugu rubripes* microRNA 21; tni-miR-21, *Tetraodon nigroviridis* microRNA 21; pol-miR-21, *Paralichthys olivaceus* microRNA 21.

The 84 conserved miRNA families and 7 miRNAs (mir-459, -462, -722, -724, -726, -727, and -731) identified in the Japanese flounder were sorted into 5 groups based on their phylogenetic distributions (Figure 5A). The 5 families (mir-1, -34, -9, -124 and let-7) are present in mammals, birds, amphibians, fish, insects and nematodes; 13 families are present in mammals, birds, amphibians, fish and insects, but not nematodes; 6 families are only present in mammals, birds, amphibians and fish; the seven miRNAs (mir-459, -462, -722, -724, -726, -727, and -731) are only identified in fish; and the remaining miRNA families are vertebrate-specific but three families (mir-456, -460, and -458) are absent in human (*Homo sapiens*) and mouse (*Mus musculus*) and 8 miRNAs are restricted to fish. The 3 miRNAs families (mir-147, -449, and -150) were first detected in fish. Although the 84 flounder miRNA families are highly conserved throughout different animal taxa, there are pedigree specific sequence substitutions in most of these families. In spite of the short sequences of mature miRNAs, the major clades are well divided due to substitutions. Fish and other vertebrates can be easily separated according to sequence differences in their miR-21, which showed the different sequence features of conserved miRNAs in different clades (Figure 5B). Scanning miRNA families, we found a family, mir-29a, by which Japanese flounder could be separated from other fish (Figure 5C). The results indicated that miRNAs might play an important role in species evolution, and nucleotide substitutions in mature miRNAs might cause diversity of miRNA regulating gene expression in animal developmental processes.

## Discussion

Using a high-throughput Solexa sequencing approach, we identified 140 conserved miRNAs and 57 miRNA*s and analysed expression profiles of these miRNAs during metamorphosis of Japanese flounder, a species without a completely sequenced genome. The reads of these miRNAs sequences ranged from 4 to 76788, indicating that not only the high expression of miRNAs but the low expression of miRNAs was identified by Solexa sequencing at the flounder metamorphic stage (Figure S1 and Figure S2). So, for studying small RNAs, Solexa sequencing is more accurate and efficient approach than the traditional cloning method by which 23 miRNAs were identified at the metamorphic stage of flounder [29]. In our studies, Japanese flounder miRNAs occupied nearly half of fish miRNAs in miRBase 13.0 shared by other animals, which will provide us with a better understanding of the evolution of species. We found that there are a few species-specific nucleotide substitutions in the ‘non-seed’ sequence regions in the bulk conserved miRNAs families. For example, the mir-21 sequences in the mammals and birds are the same, but a different mir-21 sequence in the fish species (Figure 5B). In general, these conserved substitutions occur apart from the ‘seed’ sequence [31], so some highly conserved miRNA families which exist only in some special species can also be ‘species-specific’. The ‘seed’ sequence is important for mRNA target recognition. However, it alone is not plenitudinous for miRNA-target interaction [17]. These findings will contribute to the understanding of the diversity of miRNA regulating gene expression in different biological processes.

A large amount of conserved miRNA*s were discovered in the Japanese flounder. These miRNA*s had the very high sequence similarity in animal species. Some of these miRNA*s were similar to the known miRNA*, others differed from the known miRNA* by one to three nucleotides. The function of miRNA* have been often neglected in previous studies because these sequences are usually regarded as important primarily for maintaining precursor secondary structure [1],[36]. A recent study showed that the inhibitory activity of miRNA* species is required in both cultured cells and transgenic animals [36]. Our results indicated that miRNA* could play a possible role in regulating gene expression during Japanese flounder metamorphosis, which was previously reported in flies [36].

Some miRNAs were comparatively conserved, not just in their sequences but also in their function on regulating gene expression in different animal species. In our studies, a number of key miRNAs identified in the Japanese flounder were shared with other vertebrates or invertebrates and might modulate diverse cellular signal transduction pathway in complex tissues and organs. The let-7 family, a heterochronic miRNA, is phylogenetically conserved in diverse bilaterians but not in sponge or cnidarians, and has a conserved function in heterochronic developmental regulation [23], [37]–[38]. MiR-183, miR-184 and miR-96, which are characteristic of sensory organs in vertebrates, were also identified in the Japanese flounder. Although the detailed function of these miRNAs needs to be further explored, it is intriguing to speculate that they contributed greatly to the evolution of complex animal organs. Some miRNAs were highly expressed at metamorphic stage of Japanese flounder, indicating that they might be necessary for natural metamorphosis.

With *ab initio* predictive methods, many non-conserved or species-specific miRNAs have been discovered and been experimentally validated in viruses and human [39]–[40]. In our study, using miPred with an *ab initio* predictive approach for identifying miRNA precursors, we did not discover novel flounder-specific miRNAs. In the absence of genome sequence data, it is difficult to identify new species-specific miRNAs.

Using miRNA microarray, we examined expression patterns of flounder miRNAs at two different metamorphic stages (17 and 29 dph), and obtained 102 conserved miRNAs that have been identified by Solexa sequencing (Figure S4). This further validated these miRNAs identified by Solexa sequencing. Then, we identified 66 miRNAs that were differentially expressed between 17 and 29 dph larvae (Table S4). Using Quantitative real-time PCR assay, we also found that the miRNAs (mir-22a, -10d, -10c and -7) were differentially expressed at four different developmental stages (Figure 3C). The results indicated that these miRNAs might play an important role in diverse biological processes of larval to juvenile Japanese flounder. For example, mir-1, -133, and -206 are muscle-specific miRNAs, which play a key role in regulating differentiation and proliferation of myoblasts [41]–[42]. In this study, they were differentially expressed between 17 and 29 dph during metamorphosis, indicating that they might play an important role in muscle development of metamorphic flounder, because muscular structure and function dramatically change at the metamorphic stage [21]. Previous studies have shown that mir-25 regulates pigmentation by targeting the transcription factor *mitf* in alpaca (*Lama pacos*) skin melanocytes [43]. Mir-203, a skin-specific microRNA, is up-regulated in psoriasis and regulated by keratinocyte differentiation [44]. At the present research, these two miRNAs were differentially expressed in the two different metamorphic stages of Japanese flounder, indicating that they might regulate flounder skin development and chromogenesis, because flounder skin pigment apparently changes during metamorphosis [45]–[46]. Analysis of flounder miRNA expression would be helpful for further understanding of the functions of miRNAs in flounder metamorphic development, which could help us further explain the complex genetic network that controls processes of flounder metamorphosis and provid insight into metamorphic changes.

In conclusion solexa sequencing provides an accurate and efficient approach for studying small RNAs in the Japanese flounder, as well as other species. We discovered a large number of miRNAs during metamorphosis in the Japanese flounder. We further analyzed the evolutionary path of the flounder miRNAs, indicating the significance of miRNAs in animal evolution. We found numerous differentially expressed miRNAs at two metamorphic stages of Japanese flounder, which provides the basis for future analysis of miRNA function in Japanese flounder metamorphic development and gives us clues for further studies of the mechanisms of Japanese flounder metamorphosis.

## Materials and Methods

### Experimental animal collection and RNA isolation

Japanese flounder larvae were provided by the Beidaihe Center Experiment Station, Chinese Academy of Fishery Sciences, Hebei, China. Larvae (13, 17, 19, 21, 23, 29, 31, 33, 36 and 42 days post hatching (dph)) were collected and stored in RNAstore Reagent (Tiangen, Beijing, CA, China). The metamorphic stages of Japanese flounder were defined as described by Minami [47]. Under this definition, larvae of 17, 21, 29, 36, and 42 dph are represented as stage D, Stage E, Stage G, Stage H, and Stage I of Japanese flounder metamorphic development, respectively. Total RNA was extracted separately from the whole body of above ten-stage larvae (four larvae each stage) with TRIzol reagent (Invitrogen, Carlsbad, CA, USA) according to the manufacturer's instructions. RQ1 RNase-Free DNase (Promega, Madison, WI, USA) was used to remove genomic DNA contamination. The quality and integrity of RNAs were examined using an Agilent 2100 Bioanalyzer. Then, we respectively took equal amounts of nine-stage total RNAs (17, 19, 21, 23, 29, 31, 33, 36 and 42 dph) and pooled them together for Solexa sequencing. All animal protocols were approved by the Review Committee for the Use of Animal Subjects of Shanghai Ocean University.

### Solexa sequencing of Japanese flounder small RNAs

Small RNA fragments (14–30 bases) were isolated from the total RNA pool with a Novex 15% TBE-Urea gel (Invitrogen). The purified small RNAs were then ligated with 5′ adaptors (Illumina, San Diego, CA, USA). To remove these unligated adaptors, the ligation products (40–60 bases in length) were gel purified on a Novex 15% TBE-Urea gel. Subsequently, a 3′ adaptor (Illumina) was ligated to the 5′ ligation products. After gel purification on a Novex 10% TBE-Urea gel (Invitrogen), RNA fragments with adaptors at both ends (70–90 bases in length) were reversely transcribed, and the resulting cDNAs were subjected to 15 PCR cycles using the adaptor primers. The amplification products (around 90 bases) were excised from a 6% TBE-Urea gel (Invitrogen). The purified cDNAs library was used directly for cluster generation and sequencing analysis by an Illumina/Solexa G1 sequencer.

### Sequencing data analysis

Sequencing reads were extracted from the image files generated by Illumina Genome Analyzer and then processed to produce digital-quality data. The subsequent procedures performed with Solexa were summarizing data production, evaluating sequencing quality, calculating length distribution of small RNA reads and masking low quality reads and adaptor sequences. The remaining reads were analyzed by BLAST against Japanese flounder mRNAs, Rfam (ftp://ftp.sanger.ac.uk/pub/databases/Rfam) and Repbase (http://www.girinst.org/) to discard mRNA, rRNA, tRNA, snRNA, snoRNA and repeat sequences. By aligning to the Rfam database, we could annotate a variety of annotations to each sRNA sequence, such as rRNA, tRNA, snRNA and snoRNA. By aligning to the Repbase database, we could annotate repeat sequences. Subsequently, 16–26 nt non-annotated sequences were analyzed by BLAST against miRBase 13.0. Solexa sequences with identical or related (one mismatch) sequences from metazoan mature miRNAs were identified as conserved miRNAs. Then, these Solexa sequences that can form duplex-like miRNA: miRNA* pairs and map to the conserved miRNA precursors, were considered as flounder miRNA*s.

Then, the remaining sequences were searched by BLAST against pleuronectoidei ESTs (NCBI) to identify non-conserved miRNAs. For each mappable sequence, hairpin folding was evaluated by sequence analysis to identify the presence of a stem loop with 18 or more base pairs, and the folding energy was calculated by mfold [33]. The criteria used include: (1) the number of base pairs in a stem was ≥18 nt; (2) the number of allowed errors in one bulge was ≤18; (3) free energy (ΔG) was ≤−15 kCal·mol−1; (4) percentage of miRNA appearing in the stem was ≥80%; (5) length of hairpin ( up and down stem + terminal loop ) was ≥53 nts; and (6) length of the hairpin loop was ≤22 nts. If one sequence satisfies these strict criteria, this sequence is considered a candidate of the predicted miRNA precursor.

### Amplification of the miRNA precursors

The assay to amplify miRNA precursors was conducted as previously described [17]. Briefly, we extracted genomic DNA from the larvae of metamorphosis according to the manufacture's protocol and designed primers for 20 of 57 duplex-like miRNA: miRNA* pairs with Primer Premier 5.0 (Premier Biosoft International, Palo Alto, CA, USA). Corresponding fragments were amplified by PCR and the length of amplification products was detected on 3% agarose gels. Fragments between 55 and 70 nt in length were subcloned into pMD19-T vector (TaKaRa, Dalian, CA, China) for sequencing analysis.

### Quantitative real-time PCR assay

Expression levels of flounder miRNAs were confirmed as previously described [34]–[35]. Using M-MLV Reverse Transcriptase (Promega, Madison, WI, USA), four-stage RNAs (13, 21, 29, and 42 dph) and the pool RNA used in the Solexa sequencing were reverse transcribed by looped antisense primers containing random primers, respectively. Quantitative real-time PCR was conducted on MyiQ5 Real-time PCR Detection System (Bio-Rad, Hercules, CA, USA). All primers are listed in Table S1. Japanese flounder 5S rRNA was used as a control. In each assay, 10 µl of SYBR® *Premix Ex Taq*™ (TaKaRa, Dalian, CA, China), 0.4 µl of 10 µM specific forward primer, 0.4 µl of 10 µM specific reverse primer, 7.2 µl of RNase-free H_2_O and 2 µl of cDNA were incubated at 95°C for 30 seconds and followed by 40 cycles of PCR (95°C for 10 seconds, 60°C for 30 seconds). All reactions were run in triplicate. After reaction, we calculated the relative amounts of miRNAs by the comparative threshold (2^−ΔΔCt^) method [48]. Each Ct value used for the calculation was the mean obtained from each cDNA in triplicates. The data are presented as means ± SE. Statistically significant differences were examined by paired t-test. A value of *p*<0.05 was considered to be statistically significant.

### Northern blot hybridization

Approximately 15 µg of total RNA pooled as described above was loaded per lane, resolved on a 15% denaturing polyacrylamide gel, including labeled RNA oligonucleotides as size marker, and transferred electrophoretically to Hybond-N^+^ membranes (Amersham Life Sciences, England). Membranes were UV cross-linked and baked for 1 h at 80°C. DNA oligonucleotides reverse complement to miRNA sequences were end-labeled with [γ-^32^P] ATP with T4 polynucleotide kinase (NEB, Beijing, CA, China). Membranes were prehybridized at 40°C for 4 h in hybridization buffer (0.36 M Na_2_HPO_4_, 0.14 M NaH_2_PO_4_, 7% SDS and 1 mg of sheared, denatured, salmon sperm DNA) and hybridized at 40°C overnight with 20 pmole of a γ-[^32^P]-ATP radiolabeled probe. After hybridization, membranes were washed twice with 0.5×SSC, 0.1% SDS at 40°C for 10 minutes and once with 0.1×SSC, 0.1% SDS at 40°C for 5 minutes. Membranes were briefly air-dried and wrapped with Saran Wrap. Images were acquired with a Molecular Image FX machine (Bio-Rad).

### MiRNA microarray analysis

Total RNA (5.0 µg) from 17 dph and 29 dph larvae were size fractionated with a YM-100 Microcon centrifugal filter (Millipore Corp, Billerica, MA), and the isolated small RNAs (<300 nt) were 3′- extended with a poly (A) tail using poly (A) polymerase. An oligonucleotide tag was then ligated to the poly (A) tail for later fluorescent dye staining. Hybridization was performed overnight on a μParaflo microfluidic chip (LC-Bio, Hangzhou, China) with a micro-circulation pump (Atactic Technologies) [49]–[50]. On the microfluidic chip, each detection probe consisted of a chemically modified nucleotide coding segment reverse complementary to a target miRNA (Japanese flounder conserved miRNAs and other fish conserved miRNAs) and other RNAs (control and Japanese flounder miRNA candidates). The hybridization melting temperatures were balanced by chemical modifications of the detection probes. Hybridization was performed using 100 µL 6×SSPE buffer (0.90 M NaCl, 60 mM Na_2_HPO_4_, 6 mM EDTA, pH 6.8) containing 25% formamide at 34°C. After hybridization, fluorescence labeling was detected using tag-specific Cy5 dyes. Hybridization images were collected with a laser scanner (GenePix 4000B, Molecular Device) and digitized by Array-Pro image analysis software (Media Cybernetics). Finally, hybridization signals were detected and quantified, and data were analyzed by first subtracting the background and then normalizing the signals with a cyclic LOWESS filter (Locally-weighted Regression) [51]. A miRNA detection signal threshold was defined as 32 after removal of the maximal signal level in the background. All data is MIAME compliant and the raw data has been uploaded to Gene Expression Omnibus.

## Supporting Information

Figure S1 **Frequency of Japanese flounder conserved miRNAs at the metamorphic stage.**
(TIF)

Figure S2 Frequency of Japanese flounder conserved miRNA*s at the metamorphic stage.
(TIF)

Figure S3 **Stem-loop structures of miRNA precursors in Japanese flounder.** The red letters represent the mature miRNAs, and the blue ones represent its miRNA*.
(TIF)

Figure S4 **Expression profiles of miRNAs at two metamorphic stages (17 and 29 dph) by hierarchical clustering.** Red indicates that a gene is highly expressed at the stage, whereas green indicates the opposite.
(TIF)

Table S1 **Primers used in this study for RT-PCR and Quantitative real-time PCR.**
(XLS)

Table S2 **Japanese flounder conserved miRNAs during metamorphosis.**
(XLS)

Table S3 Japanese flounder conserved miRNA*s during metamorphosis. The red letters represent mismatched base, the green labeled miRNA* indicate that they need further validate.
(XLS)

Table S4 **Differential expression of Japanese flounder conserved miRNAs between 17 and 29 dph.**
(XLS)
