# Supplementary figures and images for: Identification and Differential Expression of MicroRNAs during Metamorphosis of the Japanese Flounder (Paralichthys olivaceus)
Source: PLoS One. 2011 Jul 27;6(7):e22957. doi: 10.1371/journal.pone.0022957 (PMC3144956; doi:10.1371/journal.pone.0022957)

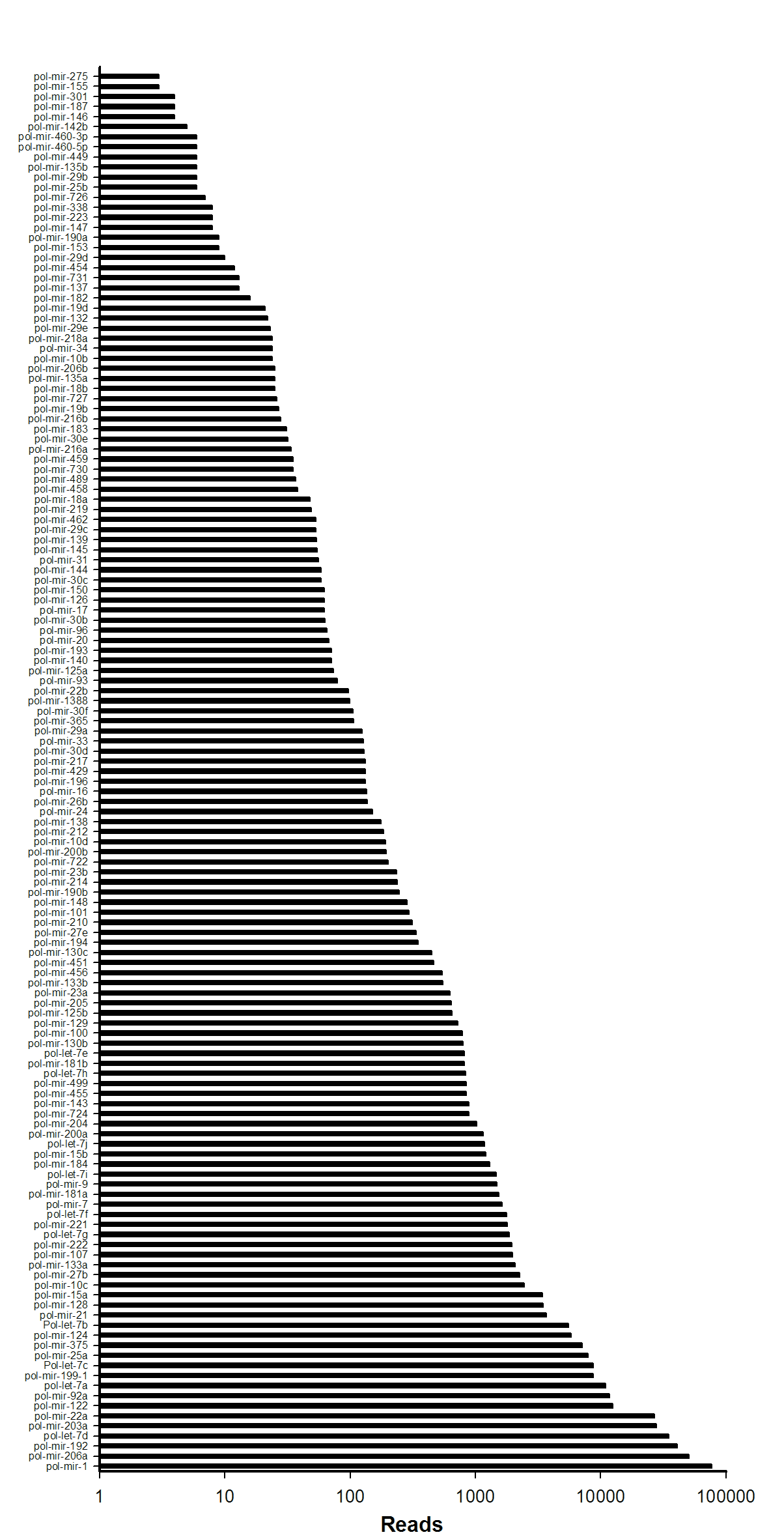

Supplement: Figure S1 — Frequency of Japanese flounder conserved miRNAs at the metamorphic stage. (TIF) [file pone.0022957.s001.tif]

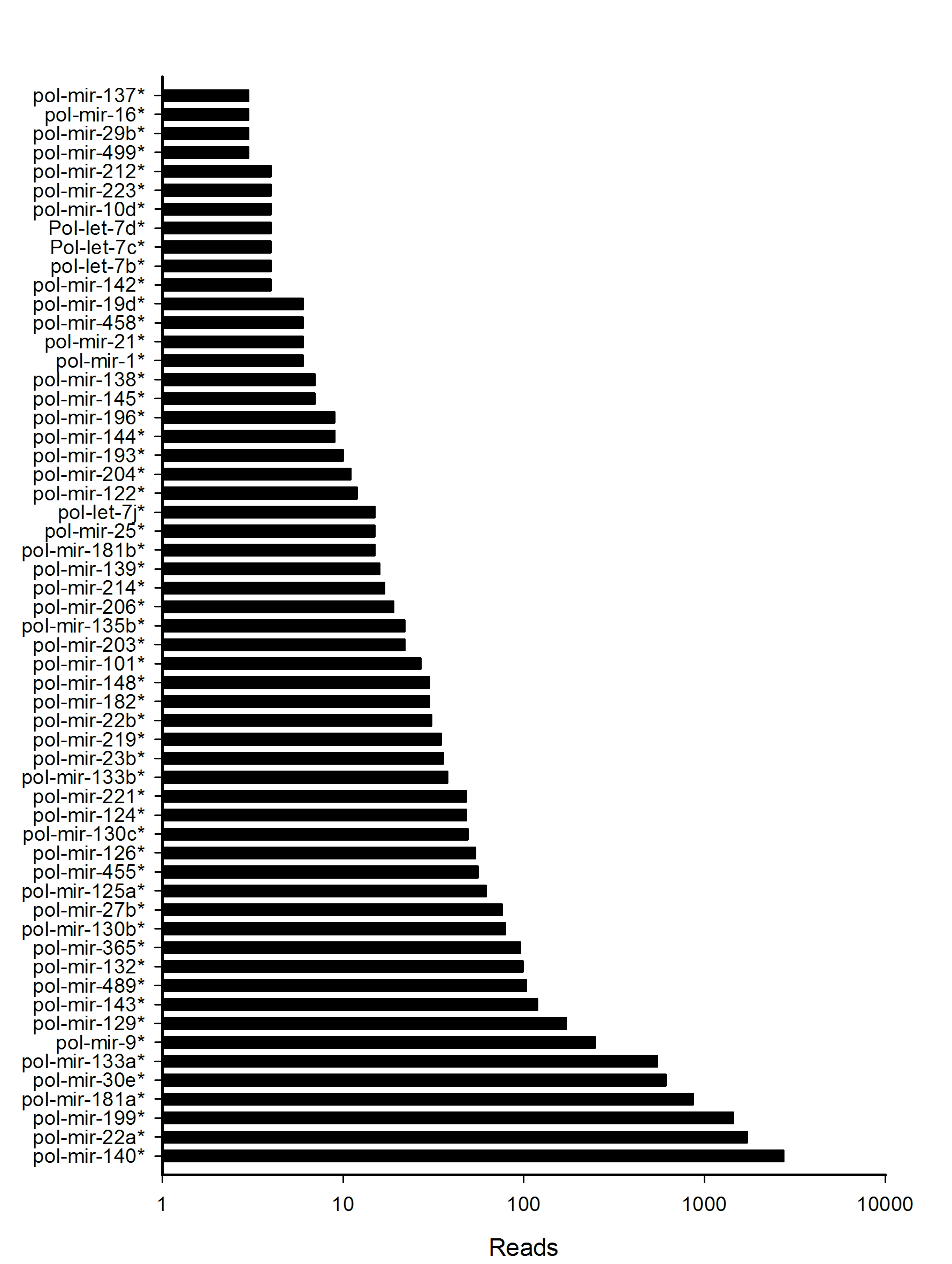

Supplement: Figure S2 — Frequency of Japanese flounder conserved miRNA*s at the metamorphic stage. (TIF) [file pone.0022957.s002.tif]

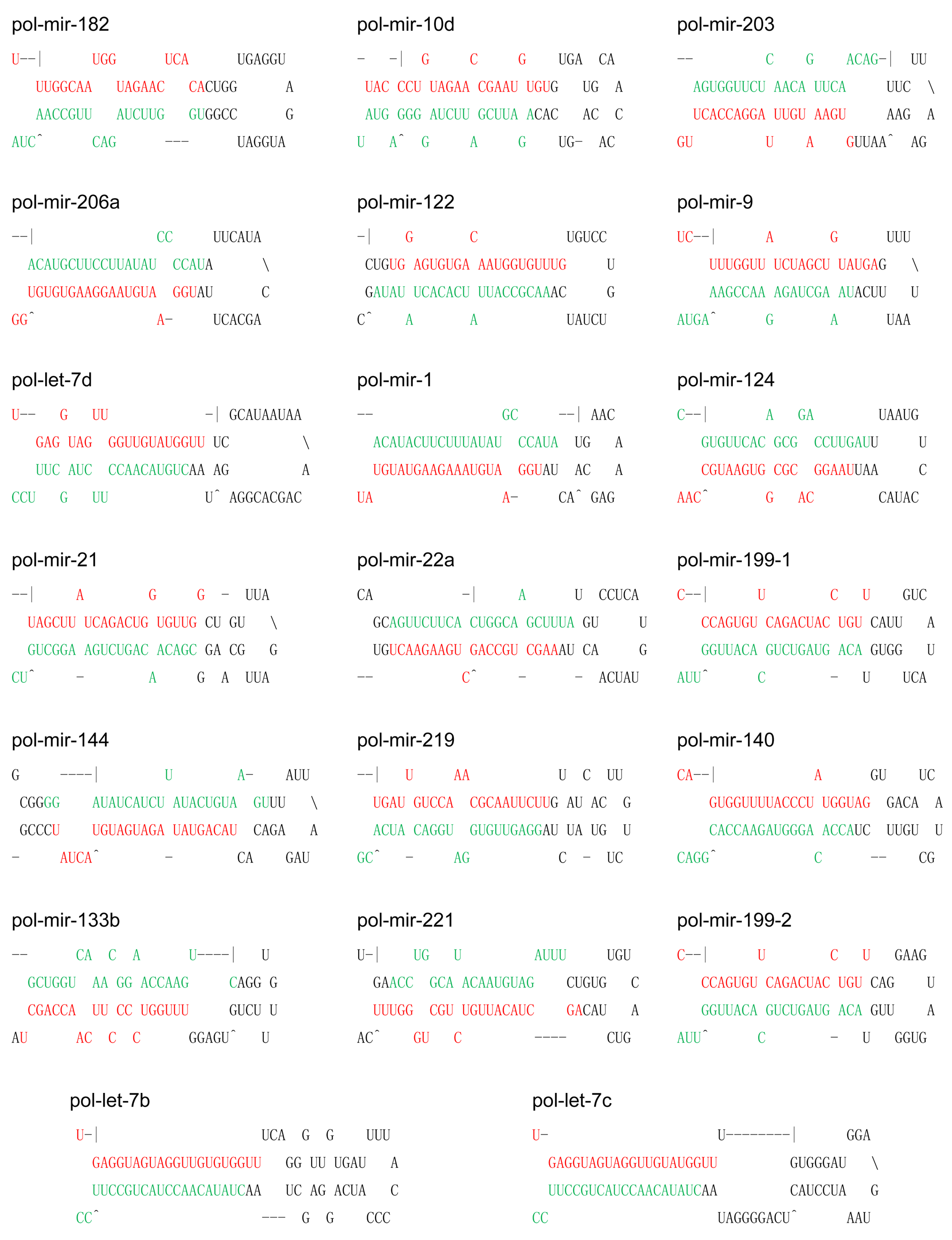

Supplement: Figure S3 — Stem-loop structures of miRNA precursors in Japanese flounder. The red letters represent the mature miRNAs, and the blue ones represent its miRNA*. (TIF) [file pone.0022957.s003.tif]

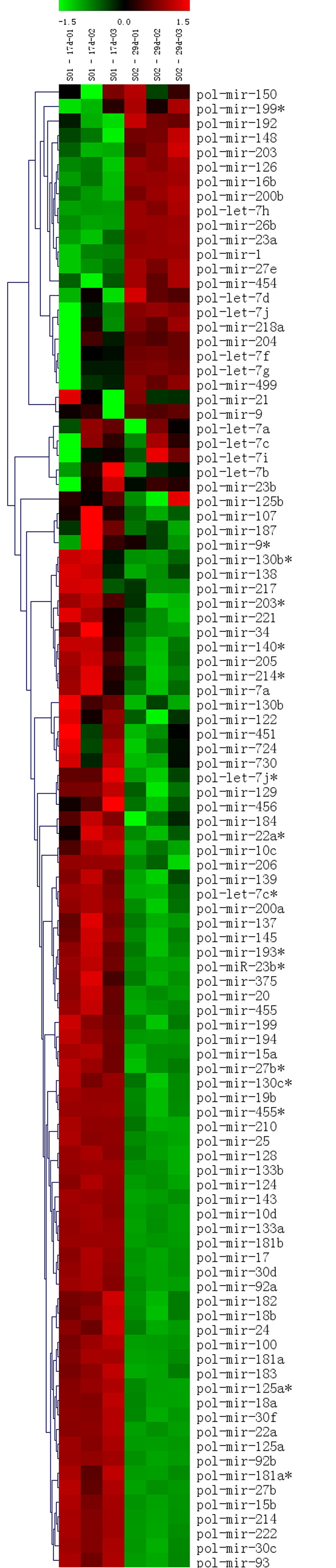

Supplement: Figure S4 — Expression profiles of miRNAs at two metamorphic stages (17 and 29 dph) by hierarchical clustering. Red indicates that a gene is highly expressed at the stage, whereas green indicates the opposite. (TIF) [file pone.0022957.s004.tif]
